# Demethylation of *MAGE* promoters during gastric cancer progression

**DOI:** 10.1038/sj.bjc.6601600

**Published:** 2004-02-17

**Authors:** T Honda, G Tamura, T Waki, S Kawata, M Terashima, S Nishizuka, T Motoyama

**Affiliations:** 1 Department of Pathology, Yamagata University School of Medicine, Yamagata, Japan; 2 Department of Internal Medicine, Yamagata University School of Medicine, Yamagata, Japan; 3 Department of Surgery, Fukushima Medical University, Fukushima, Japan; 4 Laboratory of Molecular Pharmacology, National Cancer Institute, National Institutes of Health, Bethesda, MD, USA

**Keywords:** gastric cancer, *MAGE*, demethylation

## Abstract

Melanoma antigen (MAGE)-encoding genes are expressed in various tumour types via demethylation of their promoter CpG islands, which are silent in all non-neoplastic tissues except for the testis and placenta. The clinicopathological significance of demethylation of *MAGE* genes in gastric carcinoma is not known. We investigated the promoter methylation status of *MAGE-A1* and *-A3* in 10 gastric cancer cell lines and in surgical specimens from 84 gastric cancer patients by methylation-specific PCR (MSP). Expression of *MAGE-A1* and *-A3* in the 10 gastric cancer cell lines was also investigated by RT–PCR. Any correlation between the methylation status of the *MAGE* promoters and clinicopathological characteristics of the gastric cancer patients was then assessed. Eight of the 10 gastric cancer cell lines showed demethylation of both *MAGE-A1* and *-A3*, and the remaining two cell lines did either of *MAGE-A1* or *-A3*. Expression of *MAGE-A1* and *-A3* was confirmed in seven and nine of the 10 gastric cancer cell lines, respectively. The *MAGE-A1* and *-A3* promoters were demethylated in 29% (25 out of 84) and 66% (56 out of 84) of the gastric tumour specimens, respectively. Demethylation of both *MAGE-A1* and *-A3* promoters (*n*=22) was found more frequently in gastric cancer patients in advanced clinical stages (*P*=0.0035), and these patients also exhibited a higher incidence of lymph node metastasis (*P*=0.0007) compared to those patients without demethylation (*n*=25). Furthermore, demethylation patients tended to have a worse prognosis, although this difference was not statistically significant (*P*=0.183). Demethylation of *MAGE-A1* and *-A3* occurs during progressive stages of gastric cancer, and may be associated with aggressive biological behaviour of gastric cancer.

Epigenetic alterations, including hypermethylation of promoter CpG islands and histone deacetylation of tumour suppressor and tumour-related genes (Choi *et al*, 2001; Jones and Takai, 2001; Kim *et al*, 2001; Goodman and Watson, 2002; Jones and Baylin, 2002) as well as global DNA hypomethylation (Eden *et al*, 2003; Gaudet *et al*, 2003; Lengauer, 2003), have been recognised as important contributors to carcinogenesis in humans. Global DNA hypomethylation has been observed in carcinomas of the breast, liver, and colon, and is considered to occur in the early stages of tumour development (Goelz *et al*, 1985; Cravo *et al*, 1996; Narayan *et al*, 1998; Lin *et al*, 2001; Bariol *et al*, 2003). However, little is known about promoter hypomethylation of specific genes such as oncogenes and growth-related genes, with the exception of the association between demethylation and increased expression of c-*abl*, c-*myc*, *Ha-ras*, and *raf* (Cheah *et al*, 1984; Weitzman *et al*, 1989; Sharrad *et al*, 1992; Counts and Goodman, 1994).

Human melanoma cells express antigens that are recognised by cytolytic T lymphocytes derived from the blood of tumour-bearing patients or from tumour-infiltrating lymphocytes (Boon *et al*, 1994). A number of such antigens are encoded by genes of the *MAGE* family (Van der Bruggen *et al*, 1991; Lucas *et al*, 2000). A total of 19 *MAGE* genes are located on chromosome X (Lucas *et al*, 2000), and are expressed in other tumours, including gastric cancer, in addition to melanoma (Van der Bruggen *et al*, 1991; Brasseur *et al*, 1992; Chambost *et al*, 1993; Inoue *et al*, 1995a,1995b; Gotoh *et al*, 1998; Takahashi *et al*, 1998). Although the functions of the various MAGE proteins remain to be elucidated, *MAGE* gene expression is known to be activated by promoter demethylation in a similar manner as the oncogenes and growth-related genes described above (De Smet *et al*, 1996). These genes are silent in normal tissues except for the testis and placenta (De Plaen *et al*, 1994; De Smet *et al*., 1994), and may be targets for future cancer immunotherapies (Marchand *et al*, 1999; Jang *et al*, 2001).

In the present study, we investigated the promoter methylation status of *MAGE-A1* and *-A3*, which were the most frequent targets for immunotherapy, in gastric cancers, and analysed the correlation between the *MAGE-A1* and *-A3* methylation status and clinicopathological parameters of gastric cancer patients, including event-free survival.

## MATERIALS AND METHODS

### Gastric cancer cell lines

We have investigated 10 gastric cancer cell lines with variable histologies that were cultured under appropriate conditions in our laboratory: MKN1, an adenosquamous cell carcinoma; MKN7, a well-differentiated adenocarcinoma; MKN28 and MKN74, moderately differentiated adenocarcinomas; MKN45 and KWS-I, poorly differentiated adenocarcinoma; KATO-III, a signet-ring cell carcinoma; TSG11, a hepatoid carcinoma; and ECC10 and ECC12, endocrine cell carcinomas.

### Primary gastric cancers

In total, 84 pairs of cancerous and noncancerous gastric tissues (51 differentiated and 31 undifferentiated carcinomas; 25 early-stage carcinomas that demonstrated a depth of invasion limited to the submucosa and 57 advanced stage carcinomas) were surgically obtained from 84 gastric cancer patients. These tissues were immediately frozen and stored at −80°C until analysis. All patients received a median of 36.7 months of follow-up care (range, 1–77 months). Signed informed consent was obtained from every patient to allow the use of biological materials for biological studies.

### DNA extraction

DNA was extracted from 10 gastric carcinoma cell lines and 84 primary gastric cancers and their corresponding noncancerous gastric tissues with SepaGene (Sanko-Junyaku, Tokyo, Japan).

### RNA extraction

Total RNA was isolated from 10 gastric carcinoma cell lines with the TRIZOL reagent (Gibco BRL, Life Technologies, Gaithersburg, MD, USA).

### Bisulphite modification and methylation-specific polymerase chain reaction (MSP)

Treatment of DNA samples with sodium bisulphite converts all unmethylated cytosines to uracils and does not affect methylated cytosines. Briefly, 2 *μ*g of genomic DNA were denatured with sodium hydroxide and modified by sodium bisulphite. The samples were then purified using Wizard DNA purification resin (Promega, Madison, WI, USA), treated with NaOH, recovered in ethanol, and resuspended in 30 *μ*l of distilled water. Amplification was achieved in a 20 *μ*l reaction volume containing 2 *μ*l of GeneAmp PCR Gold Buffer (PE Applied Biosystems, Foster City, CA, USA), 1.0 mM MgCl_2_, 1 *μ*l each primer, 0.2 mM dNTPs, and 1 U *Taq* polymerase (AmpliTaq Gold DNA Polymerase, PE Applied Biosystems). After heating at 94°C for 10 min, polymerase chain reaction (PCR) was performed in a thermal cycler (GeneAmp 2400, PE Applied Biosystems) for 35 cycles, each of which consisted of denaturation at 94°C for 30 s, annealing at 54°C for 60 s, and extension at 72°C for 60 s, followed by a final 7-min extension at 72°C. A positive control (Sss-I methylase-treated DNA) and negative control (distilled water without DNA) were included for each amplification. The PCR products were separated on a 6% nondenaturing polyacrylamide gel. The following primer sets were used: MG1 M forward (5′-ATT TAG GTA GGA TTC GGT TTT C-3′) and MG1 M reverse (5′-AAA CTA AAA CGT CTT CCC GCG-3′) for the methylated *MAGE-A1* sequence; MG1U forward (5′-ATT TAG GTA GGA TTT GGT TTT T-3′) and MG1U reverse (5′-AAA CTA AAA CAT CTT CCC ACA-3′) for the *MAGE-A1* unmethylated sequence; MG3 M forward (5′-CGT TTT GAG TAA CGA GCG AC-3′) and MG3 M reverse (5′-ACT AAA ACG ACG AAA ATC GAC G-3′) for the *MAGE-A3* methylated sequence; MG3U forward (5′-TGT TTT GAG TAA TGA GTG AT-3′) and MG3U reverse (5′-ACT AAA ACA ACA AAA ATC AAC A-3′) for the *MAGE-A3* unmethylated sequence. Methylated and unmethylated PCR products of *MAGE-A1* and *-A3* in gastric cancer cell lines were sequenced. The PCR products were purified using QIA Quick PCR Purification Kit (QIAGEN, Tokyo). The purified PCR products were sequenced with the BigDye Terminator Cycle Sequencing Ready Reaction Kit (PE Applied Bioststems). Gel electrophotesis, data collection, and analysis were carried out with a Genetic Analyser (model 310, PE Applied Biosystems).

### Reverse transcription-PCR (RT–PCR)

Isolated RNA was reverse-transcribed and amplified using a ONE-STEP RT–PCR System (Gibco BRL). Primer sequences used were: MG1 forward (5′-TGT GGG CAG GAG CTG GGC AA-3′) and MG1 reverse (5′-GCC GAA GGA ACC TGA CCC AG-3′) for *MAGE-A1*; MG3 forward (5′-AAG CCG GCC CAG GCT CGG T-3′) and MG3 reverse (5′-GCT GGG CAA TGG AGA CCC AC-3′) for *MAGE-A3*; *β*-actin forward (5′-AAA TCT GGC ACC ACA CCT T-3′) and *β*-actin reverse (5′-AGC ACT GTG TTG GCG TAG AG-3′) for *β*-actin. Reverse transcription–PCR products were separated on 3% agarose gels.

### Preparation of positive control

Sss-I methylase (New England BioLabs, Inc.,. Beverly, MA, USA) was used to methylate 100 *μ*g of peripheral blood DNA, which was modified by sodium bisulphite as described above.

### Statistical analysis

Statistical comparisons were performed using Fisher's exact test. A *P*<0.05 was considered significant. Survival analysis was performed using a Kaplan–Meier curve with log rank test.

## RESULTS

### Demethylation and expression of *MAGE-A1* and *-A3* in gastric cancer cell lines

**Figure 1:**
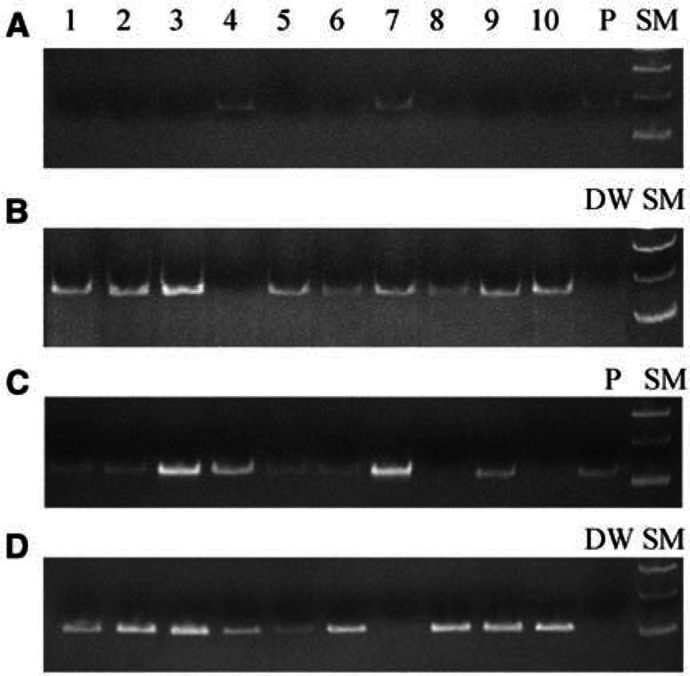
Methylation-specific PCR of gastric cancer cell lines. (**A**) Methylated-sequence-specific PCR of *MAGE-A1*; (**B**) Unmethylated-sequence-specific PCR of *MAGE-A1*; (**C**) Methylated-sequence-specific PCR of *MAGE-A3*; (**D**) Unmethylated-sequence-specific PCR of *MAGE-A3*; P, positive control; DW, distilled water; SM, size marker. Methylated *MAGE-A1* is present in lanes 4 and 7 (**A**), and demethylated *MAGE-A1* is present in all lanes except lane 4 (**B**). Methylated *MAGE-A3* is present in lanes 1–7 and 9 (**C**), and demethylated *MAGE-A3* is present in all lanes except lane 7 (**D**). Lanes: 1, MKN1; 2, MKN7; 3, MKN28; 4, MKN45; 5, MKN74; 6, KATO-III; 7, KWS-I; 8, TSG11; 9, ECC10; and 10, ECC12.

The demethylation status of *MAGE-A1* and *-A3* was determined in nine of the 10 cell lines; it was not determined for *MAGE-A1* in line MKN45 or for *MAGE-A3* in line KWS-I (Figure 1

). Both the methylated and demethylated alleles of *MAGE-A1* were present in KWS-I, and the methylated and demethylated alleles of *MAGE-A3* were present in MKN1, MKN7, MKN28, MKN45, MKN74, KATO-III, and ECC10.

**Figure 2:**
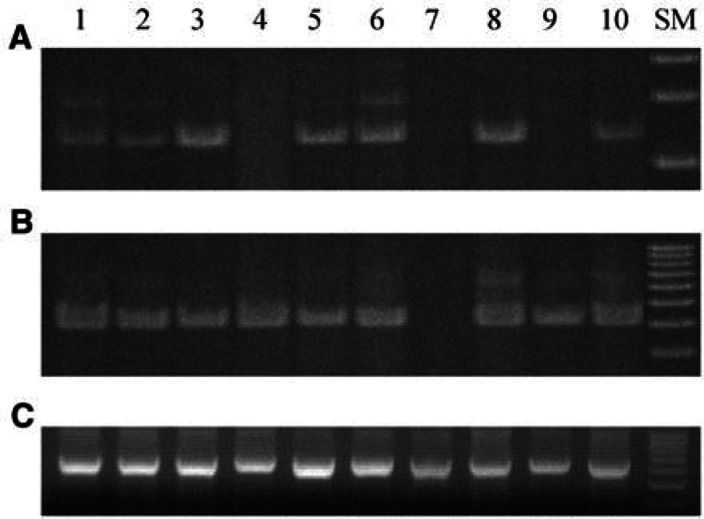
Reverse transcription–PCR of gastric cancer cell lines; (**A**) RT–PCR of *MAGE-A1*; (**B**) RT–PCR of *MAGE-3*; (**C**) RT–PCR of *β*-actin; SM, size marker. *MAGE-A1* mRNA is not present in lanes 4, 7, or 9 (**A**). *MAGE-A3* mRNA is not present in lane 7 (**B**). *β*-actin serves as an internal control (**C**). Lanes: 1, MKN1; 2, MKN7; 3, MKN28; 4, MKN45; 5, MKN74; 6, KATO-III; 7, KWS-I; 8, TSG11; 9, ECC10; and 10, ECC12.

Expression of *MAGE-A1* was confirmed in seven cell lines (MKN1, MKN7, MKN28, MKN74, KATO-III, TSG11, and ECC12), but not in the remaining three lines (MKN45, KWS-I, and ECC10), and expression of *MAGE-A3* was confirmed in all lines except KWS-I (Figure 2

). We have confirmed the methylated or unmethylated status of each *MAGE* promoter in all the gastric cancer cell lines by direct sequencing of MSP products (data not shown). Thus, promoter methylation of *MAGE-A1* and *-A3* directly correlates with their expression, except for *MAGE-A1* in line ECC10.

### Demethylation of *MAGE-A1* and *-A3* in primary gastric cancers and corresponding non-neoplastic gastric tissues

**Figure 3:**
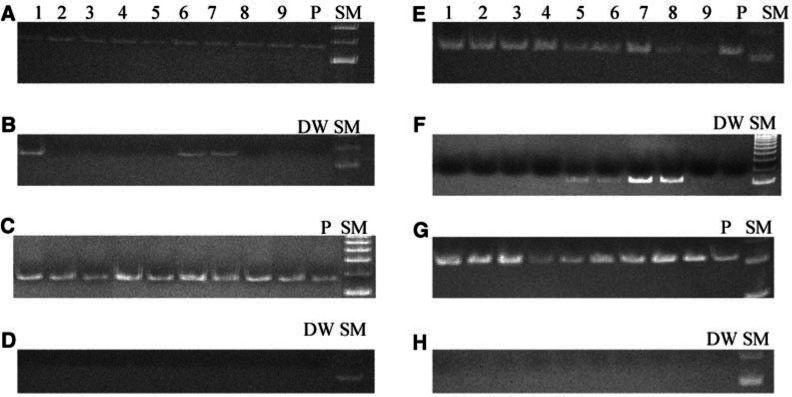
Methylation-specific PCR of primary gastric cancer specimens and their corresponding non-neoplastic gastric tissues; (**A**) methylated-sequence-specific PCR of *MAGE-A1* in gastric cancer specimens; (**B**) unmethylated-sequence-specific PCR of *MAGE-A1* in gastric cancer specimens; (**C**) methylated-sequence-specific PCR of *MAGE-A1* in non-neoplastic gastric tissues; (**D**) unmethylated-sequence-specific PCR of *MAGE-A1* in non-neoplastic gastric tissues; (**E**) methylated-sequence-specific PCR of *MAGE-A3* in gastric cancer specimens; (**F**) unmethylated-sequence-specific PCR of *MAGE-A3* in gastric cancer specimens; (**G**) methylated-sequence-specific PCR of *MAGE-A3* in non-neoplastic gastric tissues; (**H**) unmethylated-sequence-specific PCR of *MAGE-A3* in non-neoplastic gastric tissues; P, positive control; DW, distilled water; SM, size marker. Methylated *MAGE-A1* and *-A3* is present in all lanes (**A**, **C**, **E**, and **G**). In gastric cancer specimens, demethyled *MAGE-A1* and *-A3* are present in lanes 1, 6, and 7 of (**B**) and in lanes 5-8 of (**F**), respectively, whereas none of the non-neoplastic gastric tissues exhibit demethylation of *MAGE-A1* or *-A3* (**D** and **H**). Lanes: 1, M244; 2, M245; 3, M246; 4, M248; 5, M251; 6, M254; 7, M256; 8, M257; and 9, M262.

Demethylation of *MAGE-A1* and *-A3* was detected in 29% (25 out of 84) and 66% (56 out of 84) of gastric cancer samples and in 0% (0 out of 84) and 7% (6 out of 84) of their corresponding non-neoplastic gastric tissues, respectively (Figure 3

). Demethylation of both *MAGE-A1* and *-A3* was detected in 26% (22 out of 84) of the samples and of either of the genes in 44% (37 out of 84). In the remaining samples (29%, 25 out of 84), both promoters remained methylated.

### Correlation between demethylation of *MAGE* promoters and clinicopathological parameters

Gastric cancer patients who exhibited demethylation of both the *MAGE-A1* and *-A3* promoters (*n*=22) were at a more advanced clinical stage (*P*=0.0035). and had a higher incidence of lymph node metastasis (*P*=0.0007) compared with those who did not have demethylated *MAGE-A1* and *-A3* promoters (*n*=25) (Table 1

**Table 1:** Correlation of *MAGE* promoter methylation status and clinicopathological characteristics in gastric cancer patients

	**Promoter methylation status**			***P*-value**	
	**Not altered**	**One demethylated promoter**	**Two demethylated promoters**	**Not altered *vs* rest**	**Not altered *vs* two demethylated promoters**
Number of patients	25	37	22		
Age (mean) (Years)	66.8	64.6	69.1	NS	NS
*Gender*				NS	NS
M	20	25	19		
F	5	12	3		
Unknown	0	0	0		
*Stage*				0.002	0.0035
Early	15	11	4		
Advanced	10	26	18		
Unknown	0	0	0		
*Histological differentiation*				NS	NS
Differentiated	16	24	12		
Undifferentiated	9	13	10		
Unknown	0	0	0		
*Location*				NS	NS
Lower	8	15	10		
Middle	10	15	5		
Upper	5	4	6		
Unknown	2	3	1		
*Lymph node metastasis*				0.03	0.0007
Present	7	21	17		
Absent	18	16	5		
Unknown	0	0	0		

**Figure 4:**
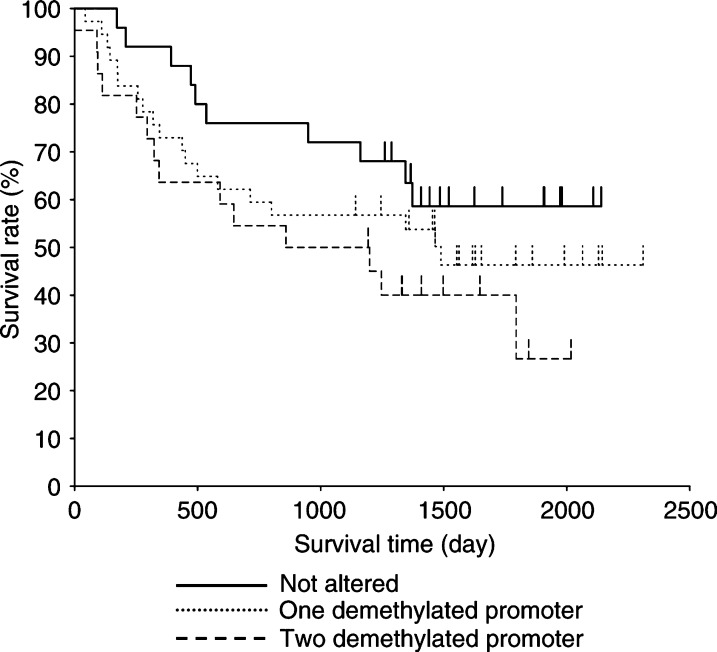
Methylation status and survival curve for gastric cancer patients. Patients in the ‘two demethylated promoter’ group tended to have a worse prognosis than patients in the ‘not altered’ group (*P*=0.183). Patients in the ‘one demethylated promoter’ group exhibited an intermediate survival time between the two.

NS=not significant by Fisher's exact probability test.

). Furthermore, patients with demethylated *MAGE-A1* and *-A3* promoters tended to have a worse prognosis, although this difference was not statistically significant by the log rank test (*P*=0.183) (Figure 4

). Patients with demethylation of only one of the two promoters exhibited biological features intermediate to those of the other two groups.

## DISCUSSION

*MAGE-A1* and *-A3* encode tumour-specific antigens that are recognised on melanoma cells by autologous cytolytic T lymphocytes (Van der Bruggen *et al*, 1991; Boon *et al*, 1994). These genes are expressed in a significant proportion of tumours of various histological types, but not in normal tissues, except for male germ line cells and placenta (De Plaen *et al*, 1994; Takahashi *et al*, 1995). Demethylation of promoter CpG islands in *MAGE* genes triggers their expression in tumour cells, whereas they are not expressed in cells in which they remain methylated (De Smet *et al*, 1996). The function of the MAGE peptides are not known, although their tumour-specific expression is clearly of great importance for immunotherapy (Marchand *et al*, 1999; Nishiyama *et al*, 2001; Sadanaga *et al*, 2001). The MAGE-A1 and -A3 peptides are expressed in 67–73% of gastric cancer cell lines (Inoue *et al*, 1995a; Li *et al*, 1996). In agreement with these data, we have demonstrated that *MAGE-A1* and *-A3* mRNA are expressed in 70 and 90% of gastric cancer cell lines, respectively. *MAGE-A1* and *-A3* mRNA has also been reported to be expressed in approximately 40% of primary gastric cancers (Inoue *et al*, 1995a,1995b; Li *et al*, 1996). However, these previous studies did not verify the methylation status of the *MAGE-A1* and *-A3* promoter CpG islands. In the present study, we showed that the methylation status of the *MAGE-A1* and *-A3* promoters almost directly correlated with their expression status in gastric cancer cell lines.

Global DNA hypomethylation is thought to occur during the early stages of tumour development in gastric and other tissues (Goelz *et al*, 1985; Narayan *et al*, 1998; Lin *et al*, 2001; Bariol *et al*, 2003). Additionally, in pulmonary carcinogenesis, demethylation of the promoter CpG islands of *MAGE* genes has been observed not only in tumours but also in the adjacent non-neoplastic lung tissues and bronchial epithelia from smokers (Jang *et al*, 2001). Therefore, *MAGE* genes may be activated prior to malignant transformation in the lung, possibly by global DNA hypomethylation (Tamura, 2002). However, we have demonstrated that *MAGE* gene promoters are demethylated more frequently in gastric cancers at advanced clinical stages. Furthermore, demethylation of *MAGE-A1* and *-A3* is quite rare in non-neoplastic gastric tissues of gastric cancer patients. In a separate study, we have confirmed that demethylation of *MAGE-A1* and *-A3* was also very rare in various organs obtained at autopsies, from various age groups (data not shown). Therefore, we hypothesise that demethylation of *MAGE* genes occurs during progressive stages of gastric carcinogenesis, probably after global DNA hypomethylation. Promoter CpG islands of several tumour suppressor and tumour-related genes are frequently methylated in both neoplastic and non-neoplastic gastric epithelia (Tamura, 2002; Waki *et al*, 2002). Hypermethylation of different genes increases with age in different organs (Waki *et al*, 2003). These results suggest that hypermethylation of promoter CpG islands occurs very early in gastric carcinogenesis, in contrast to demethylation of *MAGE* gene promoters.

Several studies have analysed *MAGE* gene expression Inoue *et al*, 1995a,1995b; Cravo *et al*, 1996; Li *et al*, 1996; Sadanaga *et al*, 2001), but none have evaluated the demethylation status of their promoters in gastric cancer. Inoue *et al* (1995a,1995b) detected *MAGE* expression in about 40% of primary gastric cancers, but failed to find any significant correlation between *MAGE* expression and clinicopathological parameters (Inoue *et al*, 1995a,1995b). In the present study, demethylation of either *MAGE-A1* or *-A3* was not significantly correlated with clinicopathological parameters, but demethylation of both genes significantly correlated with advanced clinical stage and lymph node metastasis. Furthermore, we have noticed that patients with tumours showing demethylation of both *MAGE-A1* and *-A3* tend to have a worse prognosis, although this difference was not statistically significant. In contrast, hypermethylation of the *hMLH1* gene promoter is a marker of a better prognosis (Yamamoto *et al*, 1999). No correlation has been observed between demethylation of the *MAGE* genes and hypermethylation of *hMLH1* or *p16* (data not shown).

In summary, demethylation of the *MAGE-A1* and *-A3* promoters frequently occurs during progressive stages of gastric carcinogenesis and may be associated with aggressive biological behaviour of gastric cancer.
